# Performance of older adults on MTL- BR Battery: ARE There differences in healthy aging? A pilot study

**DOI:** 10.1371/journal.pone.0329675

**Published:** 2025-08-11

**Authors:** Julia Prado Caieiro da Costa, Marcela Lima Silagi, Karin Zazo Ortiz

**Affiliations:** Department of Language, Speech and Hearing Sciences, Universidade Federal de São Paulo (UNIFESP), São Paulo, Brazil; University of Missouri Columbia, UNITED STATES OF AMERICA

## Abstract

**Objectives:**

Characterize and better understand differences that can occur in performing language tasks with advancing age in order to observe if the current norms of MTL-BR Battery obtained with individuals up 75 years of age could also be applied to old-old-individuals.

**Methods:**

80 participants, stratified into two age groups: 40 individuals aged 60–75 and 40 individuals aged 76–90 years, were submitted to an extensive language assessment by Montreal Toulouse Language Battery-Brazilian version (MTL-BR).

**Results:**

A statistically significant difference was found between both groups on the tasks of oral comprehension of sentences, narrative discourse (oral and written), written dictation, repetition of words, reading aloud of sentences and numbers, verbal naming and written numerical calculation.

**Discussion:**

The study showed that aging negatively affected performance on some language tasks. The results obtained in all tasks may be useful for comparative analyses in the clinical evaluation of old-old individuals with language complaints submitted to the MTL-BR Battery. On the one hand, subtests that typically exhibit ceiling effects in healthy populations, if found to be impaired, may clearly indicate the presence of a language disorder. On the other hand, data related to tasks that showed age-related differences should be interpreted with caution. In this sense, the data obtained on the performance of old-old individuals may guide the interpretation of the language assessment using the MTL-BR Battery or similar language assessment procedures.

## Introduction

Studies investigating aging are increasingly necessary amid the growth in life expectancy over recent decades. According to the Brazilian Institute of Geography and Statistics (IBGE), between 2012 and 2017, the Brazilian population aged 60 or older rose by 18% (4.8 million increase) to a total of 30.2 million [1]. The Pan-American Health Organization (PAHO) projects that, by 2030, 1 in 6 people will be aged 60 or over, rising to 36% by 2100 [2]

The process of aging is more than considering just the chronological age, whereby psychological, biological and social aspects also interact and influence the life of older individuals [3].

In general, with advancing age, some aspects of cognitive processing, such as processing speed, working memory, episodic memory and inhibitory function, become less effective [4,5]. Older adults can experience difficulties inhibiting irrelevant information and, consequently, may be slow to exclude from working memory information that is no longer needed to execute the task [6].

With respect to language, one of the most common age-related complaints is word finding difficulty (tip of the tongue phenomenon), where the individuals knows the word they want to say but are unable to find it. In aging, activation of phonological and orthographical representations can fail because the connections established are “one to one”, rendering these systems more vulnerable to transmission deficits. A transmission deficit in a single connection hampers recall of the phonology or orthography representations. On the other hand, semantic networks are preserved in healthy aging because they are highly interconnected, allowing a deficit in one connection to be compensated by other connections sharing the same semantic field [7].

In discourse, the reduced effectiveness of working memory seems to impact the complexity of narrative and the use of cohesive linkers [8].Oral discourse overloads working memory as the individual must remember what was said, while also planning what they are going to say next. Older adults produce a greater number of cohesive errors, such as ambiguous or incomplete references, use more pronouns, conjunctions and connectors, make pauses, reformulate words more and produce discourse containing fewer information units (units that convey information and relevance) [7–9]. There is also correlation between cognitive measures and total cohesion errors and use of lexicalization, given that better attention abilities and working memory enable more lexically cohesive discourses with fewer cohesion errors. Concerning written discourse in the context of age, the older the individual, the longer taken to produce written output [10].

Despite a number of studies showing changes in language processing with aging, many studies investigating the impact of aging on language processing have used highly specific linguistic tasks, predominantly those involving lexical access. In this context, two aspects are especially pertinent: understanding how aging changes the performance of older adults on language tasks; and establishing clinical parameters that are reliable for assessing older subjects with language deficits.

However, establishing clinical parameters in aging remains a challenge for clinicians, considering that the tests must be capable of early identification of language disorders, especially with the concern about Mild Cognitive Impairment (MCI) and Subjective Cognitive Decline (SCD), both associated with an increased risk to dementia [11,12].. This challenge becomes even greater in developing countries, due to high rates of illiteracy and low education. Since educational level and age can influence cognitive profile, it is essential to obtain tools capable of early language impairments in highly educated individuals, while also minimizing the risk of false positives among adults and elderly with low education.

Another important concern is that some measures in cognitive assessments may change over time, as different adult populations are likely to have been exposed to varying environmental stimuli and cognitive demands throughout their lifespans, potentially resulting in distinct cognitive profiles in aging [13].) Regarding cognitive functioning across the lifespan, Hartshorne and Germine [13]. observed that “not only is there no age at which humans are performing at peak on all cognitive tasks, there may not be an age at which humans are at peak on most cognitive tasks.” In this sense, the parameters used in cognitive assessments should be continuously reviewed. Furthermore, given that language is a highly complex cognitive function, it is possible that performance across various linguistic tasks can vary throughout the lifespan.

The Montreal Toulouse Language Battery – Brazilian version (MTL-BR) [14] is the only instrument for language assessment available which normative data in Brazil. Although it was designed to assess aphasia, it has many linguistic tasks that have been used not only for assessing language disorders due stroke but also for degenerative cases [15]. Language assessment is very important for diagnoses of neurodegenerative diseases, but there are still few tools for dementia and a large use of tests designed for aphasia [16,17] In fact, tests designed for aphasia can provide more data about language than the screening tests [18], although many of these batteries exhibit ceiling effects in many tasks—relative to the normative data—due to the fact that they were originally designed for PWA. This pattern is also observed in the MTL-BR Battery. The normative data for the MTL-BR Battery were established based on the performance of adults aged 18 to 75, stratified into three educational levels: 5 to 8 years of schooling, 9 to 12 years, and more than 12 years. Recent studies with illiterate and low educated individuals [19,20] showed that applying the original normative scores to such populations would result in a false-positive diagnoses of language disorders. Nevertheless, the MTL-BR Battery remains a valuable tool, as its tasks allow for the investigation and comparison of various aspects of linguistic processing.

Considering MTL-BR Battery, a previous study compared using the performance of younger healthy adults against that of older healthy adults (up to 75 years). The results revealed that age negatively impacted the tasks of structured interview, oral and written comprehension (words and sentences) and discourse, repetition, semantic verbal fluency, oral and written naming and oral textual comprehension [21]. Although the results of this study provided important information about the use of the MTL-BR in healthy populations, elderly over 75 years of age were not evaluated. Moreover, it is intriguing that previous studies showed that several-aged related linguistic abilities declines in older adults while others may be preserved or might ameliorate [22] Therefore, based on the hypothesis that there are changes in language processing associated with aging, it would be important to investigate whether such changes become more pronounced with increasing age.

Therefore, the aim of the present study was to analyze the performance of healthy older adults (aged more than 60 years) on language tasks assessed by the MTL-BR Battery.

The objectives were to examine whether the parameters established in the standardization which are used for the assessment of elderly individuals up to 75 years of age could also be applied to old-old individuals as well as tried to characterize and better understand differences that can occur in performing language tasks with advancing age.

Numerical processing and calculation tasks are part of MTL-BR Battery because it seems that there is a relationship between language and arithmetic abilities, especially in transcoding tasks (such as reading aloud number words, writing number words and Arabic numbers to dictation). Moreover, findings from a recent study [23] highlighted the importance of language in maintaining financial skills in adults and older individuals. Therefore, in addition to being part of the MTL-BR Battery, mathematical abilities also warrant further investigation.

## Methods

A prospective study was conducted at the Department of Speech, Language and Hearing Sciences of the Escola Paulista de Medicina – UNIFESP. The study was approved by the local Research Ethics Committee under permit no. 5.555.602. All volunteers signed a Free and Informed Consent Form, drafted according to recommendations of the National Board of Health pursuant to Resolution no. 466/12.

### Participants

The study sample included 80 participants, aged more than 60 years, stratified into 2 age groups: Group young old (GI) with 40 individuals aged 60−75; and Group old-old (GII), with 40 individuals aged 76−90 years. Regarding profile of GI, mean age was 65.17 (SD 4.56) years, while 25 participants (62.5%) were female and 15 (37.5%) were male. In GII, mean age was 79.78 (SD 4.6) years, whereas 30 (75%) participants were female and 10 (25%) male. No statistically significant difference was found between the groups for years of formal education (10.45 (SD ± 4.18) versus 10.25 (SD ± 4.55), p = 0.904). The years of education were controlled since cognitive functions are modified by schooling [19,21] As an inclusion criteria participants had no history of previous or current cognitive impairments, no history or diagnosis of visual or hearing deficits which could preclude conducting of the test, no history of current or previous psychiatric or neurological disorders, no use of licit or illicit psychotropic drugs (except atypical neuroleptics), and no alcohol use disorder. This information was collected using a questionnaire. In addition, all participants had to have a normal performance on the Mini-Mental State Exam (MMSE) cognitive screening, at least 7 points on the Clock Drawing Test (CDT) [24], 0 points on the Pfeffer Functional Activities Questionnaire (PFAQ) [25] and exhibit an absence of signs suggestive of depression on the Geriatric Depression Scale (GDS-15) [26] For MMSE, we adopted a Portuguese-translated version, with cut-off scores adapted to the subjects’ educational levels [27]: illiterate = 20; elementary (1 to 4 years of education) = 25; 5 to 8 years of education = 26.5; 9 to 11 years of education score. No statistically significant difference was found between the groups on the screening tests (MMSE (28.85 (SD ± 1.05) versus 28.5 (SD ± 1.24), p = 0.193; and GDS (9.45 (SD ± 0.85) versus 9.2 (SD ± 0.88), p = 0.132))

### 4.2 Procedures

All participants underwent the Brazilian version of the MTL-BR Battery.

The test was applied individually by the same examiner in a quiet room, with application time limited to 1 hour, according to the instructions contained in the application manual. The test comprises 22 subtests as outlined below:

1. Structured interview: includes 13 open-ended questions to analyze speech and auditory comprehension. 13 items with maximum score of two points each. Total score ranges from 0 to 26 points.
2. Automatic speech: assesses the ability to evoke automatisms, such as numbers, days of the week and the birthday song, evaluated for form and content. Total score ranges from 0 to 6 points.
3. Oral comprehension: measures the ability to identify images that represent words and phrases from auditory input. The task consists of a total of 19 items, five words (boards with six stimuli comprising one target and five distracters: one phonological, one semantic, one visual and two neutral) and 14 sentences. The maximum score is five points for words and 14 points for phrases, with one point for each correct answer. Total score ranges from 0 to 19 points.
4. Oral narrative discourse: evaluates the ability to tell a story from visual inputs. The task consists of describing a picture depicting a bank robbery. The narrative is analyzed for the number of words and the number of information units (IUs) produced. They IUs were considerate the informative and relevant elements that are present in an organized discourse structure and the IUs expected were: bank, robbery, thieves, guard, car, running, waiting, calling, people and money. Each IU gets point. The maximum score for IUs is 10 points.
5. Written comprehension: assesses the written comprehension of words and phrases. The task consists of a total of 13 items, five words (boards with six stimuli comprising one target and five distracters: one graphemic, one semantic, one visual and two neutral) and 8 sentences. One point is given for each correct answer, with total task score ranging from 0 to 13 points.
6. Copying: a sentence must be copied while changing the allographic form. Maximum task score is 8 points.
7. Dictation: assesses the participant’s ability to write dictated words and phrases. The task consists of 9 items (5 regular and 3 irregular words and 1 non-word) and three sentences. The maximum scores are 9 and 13 points for words and phrases respectively, with one point is given for each word written correctly, yielding a maximum score of a 22 points.
8. Repetition: measures the ability to reproduce the auditory stimuli provided. The task consists of 11 items (8 words and 3 non-words) and three sentences. The maximum scores are 11 and 22 points for words and phrases respectively, with one point for each word repeated correctly, yielding a maximum score of a 33 points.
9. Reading: assesses reading of words and phrases. The task consists of 12 items (3 irregular and 5 regular words and 3 non-words) and three sentences. The maximum scores are 11 and 22 points for words and phrases respectively, with one point for each word repeated correctly, yielding a maximum score of a 33 points.
10. Semantic verbal fluency: evaluates the ability for lexical production of words from the animals semantic category within 90 seconds. One point is given for each correctly produced word.
11. Non-verbal praxis: assesses the ability to produce isolated gestures and movement sequences involving the face and tongue, requested by the evaluator through verbal instructions. The task consists of a total of 6 items with maximum scores of 4 points each, giving a maximum total of 24 points.
12. Naming: measures lexical access using pictures that refer to nouns and verbs. Fifteen pictures are presented (12 nouns and three actions), placed on individual boards. The maximum score is 30 points, comprising 15 items with a maximum score of two points each. The criteria for scoring is incorrect answer: zero; item semantic related or description: 1 point; and correct answer: 2 points. Score on this task ranges from 0 to 30 points.
13. Object manipulation by verbal command: assesses the ability to understand simple and complex verbal commands. The individual is instructed to perform six commands given by the clinician, using physical objects (key, comb, cup, pen, and paper). The complexity of orders increases gradually. One point is given to each part of the command that is properly performed by the individual. This task is scored from 0 to 16 points.
14. Phonological verbal fluency: evaluates the number of words produced beginning with the letter M within 90 seconds. A point is given for each word produced.
15. Body part recognition and left-right orientation: assesses recognition of parts of the body and laterality orientation. The maximum score is eight points, of which four points are given for each body part (limbs) and the other four are given for the right-left orientation.
16. Written naming: fifteen pictures are presented (12 nouns and three actions), placed on individual boards. The maximum score is 30 points, comprising 15 items with a maximum score of two points each. The criteria for scoring is incorrect answer: zero; item semantic related or description: 1 point; and correct answer: 2 points. Score ranges from 0 to 30 points.
17. Oral text comprehension: assesses the ability to understand auditory input from a text read by the clinician. The individual must answer six questions orally after listening to the text (three open-ended and three closed-ended questions). A maximum of two points for each of the three open-ended questions and one point for each of the closed-ended questions Scores on this subtest range from 0 to 9 points.
18. Number dictation: assesses the ability to transcode six numbers from auditory stimuli to written form. Score ranges from 0 to 6 points.
19. Reading of numbers: assesses the ability to recognize six Arabic numerals and reproduce them orally. The task consists of six numbers. Score ranges from 0 to 6 points.
20. Written narrative discourse: involves the ability to write a story from visual input. The task consists of a picture depicting a robbery at a bakery. We analysed the number of words and the number of information units (IUs) produced. They IUs were considerate the informative and relevant elements that are present in an organized discourse structure and the IUs expected for this picture were: bakery, robbery, robbers, guard, car, running, waiting, calling and gun (one point for each word). The maximum score is 10 points for IUs.
21. Written text comprehension: assesses the ability to understand a written text. The individual must answer six questions orally after reading the text (three open-ended and three closed-ended questions). A maximum of two points for each of the three open-ended questions and one point for each of the closed-ended questions. Score on this task ranges from 0 to 9 points.
22. Numerical calculation: evaluates the ability to perform mathematical operations of addition, subtraction, multiplication and division, as well as mental simple mathematical problem. Each subtest – mental and written calculations – is scored from 0 to 6 points.

### Statistical method

After assessment of the participants and analysis of the responses obtained, the results were treated statistically.

Initially, the investigation of means, standard deviations, lower and upper limits, interquartile ranges and medians for each subtest of the MTL-BR Battery was performed for groups GI and GII. The Shapiro-Wilk test was used to check for normal distribution of data or otherwise, and also to determine the need to perform parametric or non-parametric tests when comparing performance of the two groups. Subsequently, these comparisons were performed using either the *t-*test (parametric) or Mann Whitney test (non-parametric), and the chi-square test to check the influence of advancing age on performance for each language task carried out.

The level of significance adopted for all tests was less than or equal to 5% (p ≤ 0.05). Statistical analyses were carried out using the free software R (R version 4.2.2 (2022-10-31 ucrt)).

The Shapiro-Wilk test was used to check whether the data adhered to a normal distribution, and the Mann-Whitney test applied when the p-value found was less than 0.05 The results of group comparisons were expressed according to the test employed. When the *t-*test was used, results were expressed as mean and confidence interval, followed by the significance value. For use of the Mann-Whitney test, results were expressed as median and interquartile range, followed by the significance value.

With regard to controlling for effect, in cases applying the *t*-test, the effect size calculated was Cohen´s ‘d’, expressing the number of standard deviations (SD) of difference between the results for the two groups. The following size interpretations were adopted: d < 0.2: negligible; d = 0.2–0.4: small; d = 0.4–0.8: medium; and d > 0.8: large.

On the Mann-Whitney test, effect size was calculated using the ‘*r’* statistic, where the further the value from 0 (zero), the larger the effect size. The following scale range was used: small (r > 0.1), medium (r > 0.3) and large (r > 0.5). Also, when the value of *r* was positive, the score on the test was higher in GI than GII and, when negative, the score was higher in GII than GI.

## Results

Performance data for the two groups on the MTL-BR Battery are presented in Table 1.

**Table 1:** Performance and statistical difference (p-value) between groups on subtests of the MTL-BR Battery.

Task			GI (n = 40)					GII (n = 40)			p-value (ES)
	x̄ (SD)	Mean		Q1-Q3	Min-max	x̄ (SD)	Mean		Q1-Q3	Min-max	
**1. Structured interview**	25.8 (0.61)	26		26−26	23-26	25.68 (0.66)	26		25.75-26	23-26	0.181d (r = 0.15)
**2. Automatic speech – Form**	5.9 (0.3)	6		6−6	5-6	5.92 (0.27)	6		6−6	5-6	0.701d (r = −0.04)
**Content**	5.9 (0.3)	6		6−6	5-6	5.92 (0.27)	6		6−6	5-6	0.701d (r = −0.04)
**3. Oral** **comprehension – Words**	4.92 (0.27)	5		5−5	4-5	4.85 (0.36)	5		5−5	4-5	0.296d (r = 0.12)
**Sentences**	12.9 (0.87)	13		12-13.25	10-14	12.12 (1.64)	12		11-14	8-14	0.026*d (r-0.25)
**4. Oral narrative discourse** **Number of words**	59.45 (29.23)	55.5		38-72	15-144	36.62 (16.25)	33		25.75-42.75	13-87	<0.001*d (r = 0.46)
**Time (seconds)**	43.25 (20.01)	37.11		30-53.6	12-112.3	52.98 (31.55)	50.5		31.5-64.25	14-190	0.149d (r = −0.16)
**IUs**	6.58 (1.93)	7		5.75-8	2-10	4.82 (2.34)	4		3-6.25	1-10	<0.001*d (r = 0.38)
**5. Written comprehension – Words**	5 (0)	5		5−5	5−5	4.92 (0.35)	5		5−5	3-5	0.160d (r = 0.16)
**Sentences**	7.53 (0.68)	8		7-8	6-8	7.25 (1.1)	8		7-8	4-8	0.432d (r = 0.09)
**6. Copying**	7.65 (0.8)	8		8−8	4-8	7.6 (1.01)	8		8−8	3-8	0.866d (r = −0.02)
**7. Dictation**	20.5 (2.24)	21		20-22	12-22	17.75 (4.41)	19		13.75-21	9-22	0.002*d (r = 0.34)
**8. Repetition** **Words**	10.88 (0.33)	11		11−11	10-11	10.2 (0.97)	11		9-11	8-11	<0.001*d (r = 0.41)
**Sentences**	21.85 (0.66)	22		22−22	18-22	21.82 (0.5)	22		22−22	20-22	0.471d (r = 0.08)
**9. Reading** **Words**	11.15 (1.19)	11.5		11-12	7-12	11.3 (1.04)	12		11-12	8-12	0.581d (r = −0.06)
**Sentences**	20.92 (0.35)	21		21−21	19-21	20.73 (0.55)	21		21−21	19-21	0.027*d (r = 0.25)
**10. Semantic verbal fluency**	20.92 (5.31)	22		17-23.25	8-32	19.52 (6.09)	19.5		15-23	6-36	0.277c (d = 0.25)
**11. Nonverbal praxis**	23.38 (1.19)	24		23-24	19-24	23.62 (0.93)	24		24−24	20-24	0.285d (r = −0.12)
**12. Naming**	29.52 (0.82)	30		29-30	27-30	28.73 (1.63)	29		28-30	23-30	0.010*d (r = 0.29)
**13. Objects manipulation**	15.78 (0.42)	16		16−16	15-16	15.9 (0.38)	16		16−16	14-16	0.074d (r = −0.2)
**14. Phonological verbal fluency**	14.93 (5.63)	14		11.75-17.25	7-37	14.78 (7.12)	13.5		11-18.5	4-33	0.813d (r = 0.03)
**15. Body part recognition**	4 (0)	4		–	4−4	4 (0)	4		–	4−4	–
**Left-right orientation**	3.98 (0.16)	4		4−4	3-4	4 (0)	4		4−4	4−4	0.330d (r = −0.11)
**16. Written naming**	27.5 (1.96)	28		26-29	23-30	25.85 (4.44)	28		23.75-29	14-30	0.315d (r = 0.11)
**17. Oral text comprehension**	7.72 (1.38)	8		7-9	3-9	7.05 (1.85)	7		6-9	2-9	0.113d (r = 0.18)
**18. Number dictation**	5.7 (0.69)	6		6−6	3-6	5.75 (0.49)	6		6−6	4-6	0.898d (r = 0.02)
**19. Reading of numbers**	5.97 (0.16)	6		6−6	5-6	5.78 (0.58)	6		6−6	3-6	0.026*d (r = 0.25)
**20. Written narrative -** **Total of words**	36.1 (21.38)	31		20.75-47	4-95	21.45 (12.41)	20		12.5-29.75	3-45	0.001*d (r = 0.36)
**Time**	156.84 (102.67)	125.5		93-213.5	5.54-435	83.25 (52.21)	65		54.25-103.5	15-240	<0.001*d (r = 0.43)
**Total IU**	6.35 (2.32)	7		5-8	2-10	4.45 (2.28)	4		3-6	1-10	<0.001*d (r = 0.38)
**21. Written text comprehension**	8.57 (0.96)	9		8-9	8-9	8.03 (1.42)	9		7-9	4-9	0.067d (r = 0.21)
**22. Numerical Calculation Mental**	5.28 (0.88)	5.5		5-6	3-6	4.92 (1.35)	6		4-6	2-6	0.519d (r = 0.07)
**Written**	5.28 (1.54)	6		5-6	0-6	4.62 (1.64)	5.5		3-6	1-6	0.034*d (r = 0.24)

Letters next to the p-value indicate the statistical test used: ‘c’ = Student’s t-test (effect size – ES – measured by Cohen’s d); ‘d’ = Mann-Whitney test (ES measured by r). For d: < 0.2 = negligible; 0.2–0.4 = small; 0.4–0.8 = medium; > 0.8 = large. For r: > 0.1 = small; > 0.3 = medium; > 0.5 = large. Positive r values indicate higher results in Group I (GI); negative values indicate higher results in Group II (GII). The asterisk (*) indicates statistically significant values.

The increase in age influenced the performance of individuals on some tasks of the MTL-BR Battery. Group II showed significantly worse performance in the following tasks: oral sentence comprehension, total number of words and total number of information units (IUs) in oral narrative discourse, dictation writing, word repetition, reading aloud of sentences, oral naming, number reading, total number of words and total number of IUs in written narrative discourse, and written numerical calculation. Additionally, it is noteworthy that the total time spent on producing the written discourse was shorter in Group II.

With increasing age, a decline in performance was also observed in the tasks of semantic and phonological verbal fluency, written naming, oral text comprehension, written text comprehension, and mental calculation, as well as a longer time required to produce the oral narrative discourse. However, although differences were found between the two groups, these differences were not statistically significant.

On the other hand, the tasks of guided interview, automatic language (form and content), oral comprehension of words, written comprehension of words and sentences, copying, sentence repetition, reading aloud of words, nonverbal praxis, object manipulation, body part recognition and right-left orientation, and number dictation were not influenced by age.

## Discussion

This study aimed to verify whether the normative data established for the MTL-BR Battery for individuals up to 75 years of age could be applied to old-old, or whether advancing age might lead to changes in linguistic processing thereby resulting in different patterns of performance on the test. In this sense, the main findings of this study were that advancing age was associated with a decrease in performance on some language tasks of the MTL-BR Battery in neurotypical elderly while there were no differences between groups in other tasks that are commonly easily performed for young and old individuals.

Table 1 presents the data obtained from the eighty elderly participants in this study, as well as group comparisons for all tasks. One noteworthy observation is that a ceiling effect was identified in many tasks. This effect had also been reported in the normative data, regardless of age and educational level, for some tasks in the MTL-BR Battery. Although many of these tasks are simple for neurotypical adults and elderly individuals, they may be extremely difficult for people with aphasia (PWA), for whom the test was originally developed.

Nonetheless, prior studies using the MTL-BR Battery have reported variations in performance based on sociodemographic variables such as education and age for certain tasks [10,19,20]. These findings motivated the present study, particularly given the growing increase of old-old individuals and the fact that the MTL-BR remains the only language test for adults in available in Brazil.

Moreover, in this study we observed that advancing age influenced performance of individuals on oral comprehension of phrases, total words and IU’s on oral narrative discourse, dictation, repetition of words, reading aloud of phrases, oral naming, reading of numbers, total words and IU’s on written narrative discourse and numerical written calculation, where GII showed statistically significant worse performance. These findings will be first discussed below, in an attempt to try to understand in better detail how linguistic processing can change throughout aging.

Regarding comprehension tasks, the difficulty was greater for oral comprehension of sentences, perhaps explained by the fact that the images used to represent the sentences require syntactic and visual processing. The sentences from the MTL-BR Battery are depicted in the form of drawings which have some similarities between stimuli, placing higher demands on both attention and working memory that are involved in sentences comprehension [28]. As the sentence structure becomes more complex, incorporating non-canonical or passive structures (with clauses embedded in the phrase), the demand on working memory increases [16].The subject has to store, organize and decode elements of the sentence in order to select the correct image. For written comprehension of sentences, no differences between the two groups were evident. This absence of decline in performance may be due to the maintenance of the written stimulus, allowing the individual to reprocess the sentence as many times as necessary until choosing one of the drawings, reducing the demands placed on short-term and working memory. Furthermore, the comparison between the two tasks suggests that syntactic processing, *per se*, appears to be well preserved even in old-old individuals.

Observing the oral narrative discourse task, participants produced fewer words and IUs with increasing age. Although the number of words is important for assessing persons with aphasia, it is definitely a measure that must be considerate with others. In this sense, considering MTL-BR in which the narratives produced have a brief and restricted analysis, an isolated analysis of number of words cannot predict differences in language production since a higher number of words is not necessarily linked to a better quality of narratives. In fact, personality and individual variables can influence discourse production. The IUs, by the other hand, is related to the information that must be provided by the subject. They are the very relevant elements that are present in the discourse. The IUs are single words that need to be accurate, relevant and informative to the story [29]. In the old-old group, the decrease in number of IUs seems to reflect the difficulty to word retrieval and to access specific words to describe the scene. Marini *et al* [30] found greater occurrence of semantic paraphasias among individuals aged 75–84 years on describing a single image. The authors hypothesized that the lower thematic informativeness and IUs associated with semantic paraphasias might stem from lexico-semantic processing deficits with increasing age.

With regard to task timing, participants aged >75 years took longer to complete the oral narrative discourse task, mirroring results already reported [9,10]. For written narrative discourse, the time taken on the task was shorter in the group aged >75 years, although this group used fewer words overall. Longer task time might indicate problems affecting lexical access [9], then it is possible that the shorter task time observed and the fewer words produced, appears to mirror the differences identified in previous studies. Besides, regarding narratives, during the written revision, elderly adults can have difficulty in simultaneously constructing complex sentences and their plots [28]. In our study, the narratives were well formed (coherent, grammatical and clear) and in this case, it is possible that the old-old participants just spent more time reviewing them. It is worth mentioning that on the MTL-BR Battery this is an easy discursive production task, in which an image provides visual clues of the logical and temporal sequence of events. Consequently, these factors may have facilitated the production of discourse.

Regarding the repetition task, it involves abilities of temporal ordering of sounds that starts with hearing that is a sensory system often negatively impacted by aging. Even in the absence of hearing loss, auditory abilities (central auditory processing) may be impaired [31–33]. Before repeating a stimulus, the subject must both hear and reverberate it. In the present study, the performance on repetition of words and pseudowords declined with age. During the aging process, repetition of long and low lexical frequency words, as well as pseudowords, can be negatively impacted. Difficulty repeating long words is associated with decline in working memory (phonological loop) and speech rate, since if the subject repeats slowly, there is a greater likelihood of storing less information on the target word [34,35].Moreover, low frequency words place greater demands on the motor system which, in turn, might lead to more errors. In the case of pseudowords, the closer these resemble real words, where such stimuli similarity can be found in the MTL-BR Battery, the more difficult they are to repeat. One theory behind this phenomenon is that, when pseudo and real words are phonologically similar, presentation of the pseudoword activates real words in the semantic lexicon and increases the likelihood of inhibiting correct repetition [36]. However, it interesting to note that performance on phrase repetition remained stable. In this case, the semantic association between words of the stimuli can serve to guide information processing, provide cues aiding retrieval [37,38]. These aspects allow the individual to use the context to offset and remap the stimuli heard, reverberating it for subsequent repetition [39,40]. The difficulty seen on the naming task might not be attributable to decline in vocabulary *per se*, since vocabulary and semantic knowledge are very little impacted in healthy aging [41], but rather to difficulties associated with older age, such as lexical competition and frequency. From the point at which the individual visualizes and processes the drawing in the task and activates its mental and semantic representation, a number of activations take place within the lexicon which “compete” with one another until the target word is selected. Attention, processing speed and executive functions, all of which show aging-related decline, are involved (such as inhibition) in aiding the process of selection of one of the activated items [35,41–45] and in keeping them active until naming.

For reading sentences aloud, given that rapid connected speech production is necessary, the participants may have used predictive processing of the words making up the sentence [46], given that word omission and substitution errors (particularly functional closed class) were observed. With regard to numbers, the longer and higher the number, the greater the dependence on syntactic processing, where this factor may have influenced performance [47].

A group difference was detected for written dictation. In fact, performing this task involves interactions between linguistic, spatial and perceptual (visual) systems, and depends on knowledge of the grapheme system, ability to convert phonemes into graphemes and/or to directly access the orthographic lexicon. Akin to the repetition task, written dictation also relies on auditory processing, which is negatively impacted by aging because the sounds of the words or phrases dictated must be processed in order to write them [48].Also, the effect of length must also be taken into account, since the chance of errors in written dictation increases as stimuli become longer, because they overload both the phonological loop and working memory of the grapheme buffer [49]. In the case of the lexical route, when failures occur or the written word is not stored in the orthographic lexicon, errors arise due to inadequate use of the phonological route [50,51], where older individuals are more prone to regularization errors [52].

In this study, GII had more difficulty performing written calculations, but there was no group difference for mental calculations. Mental and written calculations on the MTL differ in complexity, where mental calculations involve one digital place and written calculation two decimal places. The complexity of the calculation can impact performance [53–55]. Operations such as multiplication, which involves successive additions and recall of the times table from memory, division, which can involve successive subtractions, and subtraction, that may entail decomposition, borrowing and grouping rules, can negatively affect task performance [56]. The decline and difficulties observed on written calculation tasks may be more attributed to age-related impairments in problem comprehension and application of arithmetic knowledge [53,54,] than to loss of this knowledge, given that it is normally preserved in healthy aging [53, 55.] With increasing age, a decline in performance was also observed on the semantic and phonological verbal fluency tasks, on written naming, oral comprehension of words and text, written comprehension and mental calculations, while more time was required to produce the oral narrative discourse. However, in these tasks, the difference detected between the groups was not statistically significant.

On the phonological and semantic verbal fluency tasks, the participants encountered major difficulty producing words, likely explained by the fact this ability tends to exhibit substantial aging-related decline. In the case of phonological verbal fluency, difficulties recalling words might be associated with the vulnerability of the architecture of phonological representation, which increases the probability of failures during activation of items of phonological information, leading to the “tip of the tongue” phenomenon [7,57,58]. Regarding the semantic verbal fluency tasks, given the numerous subcategories within a semantic criterion, requiring organized hierarchical lexical retrieval, as well as suppression of competing representations, the executive functions (organization, inhibition and flexibility) and attention can become overloaded.

GI and GII scored very high on many subtests, often reaching the maximum score, although GII showed a higher standard deviation. In fact, healthy aging does not seem to affect individuals always in the same way and the inter-individual variability increases significantly [59], what seems to be confirmed by our results. This possible means that a specific ability might be preserved in one person but not in another, and not in all individuals or at the same way.

Although older participants aged over 75 years exhibited worse performance than those aged between 60–75 on some MTL-BR tasks, no differences between groups were found in structured interview, automated speech (form and content), oral comprehension of words, written comprehension of words and phrases, copying task, repetition of phrases, reading aloud of words, non-verbal praxis, object manipulation, body part recognition and left-right orientation, or number dictation. Many of these tasks are, in fact, extremely easy, and a ceiling effect has already been identified in the normative data presented in the battery.

The tasks automatic speech, recognition of body parts and left-right orientation and number dictation frequently involve knowledge that is accurately and effortlessly recalled and accessed. For example, the verbal information required in the automated language task is one of the most resistant to aging [60,61].

Non-verbal praxis involves the motor-sensory system, which is subject to decline with the effects of aging. Changes in the oral cavity can also inhibit the articulators from moving more rapidly [62,63]. The MTL-BR, however, only measures accuracy, um único movimento é pedido sem o controle do tempo, o que torna a tarefa muito fácil para os participantes neurotípicos.

Object manipulation is the least sensitive task of the MTL-BR for identifying differences, owing to the familiarity of the objects used, facilitating lexical access and performing of commands [64].

Transcoding on the copying task predominantly involves the lexical route which, through visualization of the written word, directly activates its orthographic representation and motor programming to form the letters and change the writing of one word to another [65,66]. Besides, the effects of aging may not be evident on some tasks because an underlying support network, developed and strengthened during the lifespan, involving knowledge such as semantic and verbal, was continually used, allowing older adults to devise compensatory strategies to perform certain tasks. By the other hand, an overview from our data suggests that the effects of aging appear to be more evident on tasks requiring recruitment of multiple cognitive domains [4,10,41,66,67]. Thus, considering all linguistic processing, it is possible that language is one of the most preserved cognitive function in normal aging [68].

Moreover, although we observed differences in some linguistic processing with aging, the differences between groups GI and GII identified in the tasks investigated in this study do not actually seem to interfere with the general communication of the elderly.

In the structured interview, where questions of different complexities (including opinions about situations) were asked to the elderly, no differences were observed, with the both groups of elderly almost reaching the maximum score.

Overall, despite the differences observed and previously discussed, the greatest number of modifications occurred in tasks involving reading and writing and, in relation to oral language, in tasks involving lexical access.

Thus, despite there being differences in linguistic processing with advancing age, it does not appear that such differences affect everyday communication activities, as suggested by the results of the structured interview. Studies that investigate the permanence and frequency of reading and writing habits in the elderly population can help to elucidate the greater differences between groups in reading and writing tasks in relation to those identified in oral language tasks.

### Study limitations

This study identified some differences in language processing with advancing age. Longitudinal studies focusing on the linguistic tasks that showed differences in the present study, and that also assess and monitor other cognitive domains, may contribute to a better understanding of how cognitive domains interact and how compensatory strategies that support communication are implemented. Besides, further studies exploring other variables such as years of formal education, sociolinguistic factors such as housing (nursing home versus private, in a couple or not, a situation of communicative isolation or inclusion) and socio-professional status of the subjects should be considerate

to ascertain whether these factors have a neuroprotective effect against age-related possible declines in communication in old age.

## Conclusion

The study showed that aging negatively affected performance on some language tasks from MTL-BR. These results also highlight the need for caution when interpreting the findings of language assessments of old-old individuals with language complaints, aphasia, MCI and dementias – especially those with predominant language disorders like Primary Progressive Aphasias, using the MTL-BR Battery tasks, considering the results identified in the present study. Besides, this is just a pilot study – the data obtained on the performance of healthy old-old can serve as a reference in language assessments elderly individuals using the MTL-BR Battery and perhaps for guiding the interpretation of assessment procedures similar to the language tasks performed in the present study.

## Abbreviations:

CDT: Clock Drawing Test
GDS-15: Geriatric Depression Scale
GI: Group I
GII: Group II
IBGE: Brazilian Institute of Geography and Statistics
MMSE: Mini-Mental State Exam
MTL-BR: Montreal-Toulouse Language Assessment Battery
PAHO: Pan-American Health Organization
PFAQ: Pfeffer Functional Activies Questionnaire
IU’s: InformationUnits
